# ZASP: A Highly Compatible and Sensitive ZnCl_2_ Precipitation-Assisted Sample Preparation Method for Proteomic Analysis

**DOI:** 10.1016/j.mcpro.2024.100837

**Published:** 2024-09-06

**Authors:** Xianfeng Shao, Yuanxuan Huang, Rong Xu, Qiqing He, Min Zhang, Fuchu He, Dongxue Wang

**Affiliations:** 1 State Key Laboratory of Proteomics, Beijing Proteome Research Center, National Center for Protein Sciences (Beijing), Beijing Institute of Lifeomics, Beijing, China; 2 Beijing Proteome Research Center, Beijing, China; 3 International Academy of Phronesis Medicine, Guangzhou, Guangdong, China; 4 The π-Hub Infrastructure, Guangzhou, Guangdong, China; 5 Guangzhou Laboratory, Guangzhou, Guangdong, China; 6 Department of Dermatology, Xiangya Hospital of Central South University, Changsha, Hunan, China

**Keywords:** sample preparation, proteomic, LC-MS, ZASP, incompatible detergent, SDS, Triton X-100, unbiased, sensitivity, reproducibility

## Abstract

Universal sample preparation for proteomic analysis that enables unbiased protein manipulation, flexible reagent use, and low protein loss is required to ensure the highest sensitivity of downstream liquid chromatography-mass spectrometry (LC-MS) analysis. To address these needs, we developed a ZnCl_2_ precipitation-assisted sample preparation method (ZASP) that depletes harsh detergents and impurities in protein solutions prior to trypsin digestion via 10 min of ZnCl_2_ and methanol-induced protein precipitation at room temperature (RT). ZASP can remove trypsin digestion and LC-MS incompatible detergents such as SDS, Triton X-100, and urea at high concentrations in solution and unbiasedly recover proteins independent of the amount of protein input. We demonstrated the sensitivity and reproducibility of ZASP in an analysis of samples with 1 μg to 1000 μg of proteins. Compared to commonly used sample preparation methods such as SDC-based in-solution digestion, acetone precipitation, FASP, and SP3, ZASP has proven to be an efficient approach. Here, we present ZASP, a practical, robust, and cost-effective proteomic sample preparation method that can be applied to profile different types of samples.

## Highlights

• ZASP can deplete incompatible detergents to ensure sensitive LC-MS analysis.
• ZASP occurs at room temperature and can be achieved in a simple biochemistry lab.
• This approach can be utilized for multipurpose proteomics sample preparation.
• It facilitates efficient, sensitive, and low-cost proteomics sample preparation.

## Graphical Abstract

**Figure:**
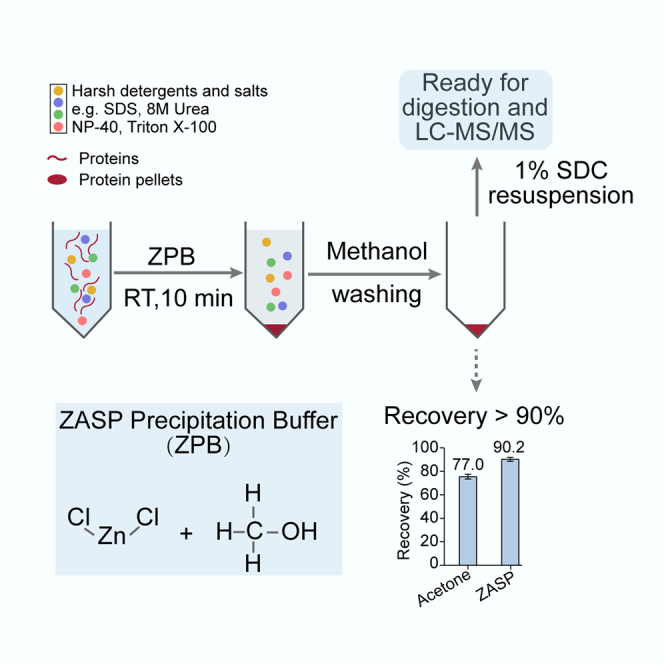

## In Brief

By combining ZnCl_2_ and methanol-induced precipitation and in-solution digestion, a practical and universal sample preparation method termed ZASP is described. ZASP connected the efficient protein solubilization by harsh detergents and the following trypsin digestion and LC-MS analysis. It shows significant advantages in processing a variety of samples.

Multistep sample preparation is a critical part of the proteomic workflow to ensure the reproducibility and comprehensiveness of liquid chromatography-mass spectrometry (LC-MS) analysis (1). Decent sample preparation is necessary for complete protein extraction and removal of impurities that interfere with chromatography, ionization, and mass spectrometer sequencing. Although detergent-free methods such as sample preparation by easy extraction and digestion (SPEED) have been developed (2), protein extraction from complex biological samples is commonly achieved via lysis buffers containing strong detergents and salts incompatible with the following steps and difficult to remove (3, 4, 5). For example, even at low levels, SDS can inhibit trypsin activity, interfere with chromatographic separation, and cause severe suppression of mass spectrometric signals (6, 7). Different protocols to remove contaminants, including in-gel digestion (8), protein precipitation in organic solvents (9), filter-aided sample preparation (FASP) (10), protein aggregation on magnetic beads) (*e.g.*, single-pot solid-phase-enhanced sample preparation (SP3)) (3, 11), and trap-based strategies (12), based on various biochemical principles have been reported. However, each of these detergent depletion methods has drawbacks, such as being labor-intensive or time-consuming, resulting in sample loss, high cost, or low throughput. An important remaining challenge is the development of a universal sample preparation method that can be used with protein lysates with different incompatible chemicals. Moreover, this method needs to be compatible with different starting materials and sample types and should be robust, reproducible, practical, and cost-effective. Although different approaches have been developed to address these challenges (3, 13), no single solution that meets all these needs is available.

Zn^2+^-based precipitation methods have been widely used for the initial fractionation of proteins based on their solubility, such as in the purification of antibodies from cell culture broth (14, 15). ZnSO_4_ and acetone aqueous solutions have been reported to rapidly recover peptides generated by trypsin and pepsin digestion prior to MS analysis (16). Zn^2+^ may bind to histidine and cysteine residues on the surface of proteins via exposed imidazole and thiol groups to form relatively stable complexes (17). In addition, as a member of the Hofmeister series, Zn^2+^ can cause protein precipitation by altering the solubility of peptides and proteins in organic solvents (18, 19, 20). Because of the stronger electrostatic interactions, Zn^2+^ is superior to other ions (e.g. Mg^2+^) in precipitating proteins from solutions (21). Inspired by these studies, we report a sample preparation method by combining ZnCl_2_ and methanol-induced protein precipitation with in-solution digestion, termed the ZnCl_2_ precipitation-assisted sample preparation method (ZASP). This procedure significantly increased the recovery of proteins from lysates with different trypsin- or LC-MS-incompatible detergents. ZASP is a simple, robust, sensitive, highly reproducible, and low-cost sample preparation procedure for proteomic analysis that can be easily implemented for various types of biological samples.

## Experimental Procedures

### Cell Culture

HEK 293T cells were cultured in Dulbecco's Modified Eagle's Medium (DMEM; Gibco) supplemented with 10% fetal bovine serum and 1% penicillin-streptomycin (Gibco) at 37 °C in an environment with 5% CO_2_. Cells were harvested at 80% confluence using 0.05% trypsin-EDTA solution and subsequently washed three times with phosphate-buffered saline (PBS). The cell pellets were then stored at −80 °C until further processing.

*Saccharomyces cerevisiae* was cultured in yeast extract peptone dextrose media at 30 °C with shaking at 200 rpm. Cells were harvested by centrifugation at 4000*g* for 5 min when the optical density at 600 nm reached approximately 1.0, followed by three washes with PBS. The cell pellets were stored at −80 °C until further processing.

*Escherichia coli* cells were cultured in Luria-Bertani (LB) medium at 37 °C with vigorous shaking. Cells were harvested during the stationary phase by centrifugation at 4000*g* for 10 min and washed twice with PBS. The cell pellets were stored at −80 °C pending protein extraction.

### Mouse Tissue Collection

All mice used in this study were bred and housed under institutionally approved conditions at the animal facility of the Beijing Proteome Research Center. All experimental procedures were conducted following approval from the Institutional Animal Care and Use Committee of the Beijing Proteome Research Center (Approval No. NCPSB-20230524-31MT) and complied with the National Institutes of Health guidelines. Mouse tissues were collected from three healthy 16-week-old C57BL/6 mice.

### Protein Extraction

In this study, lysis of most samples was performed using a 4% SDS buffer. Specifically, HEK 293T cells, yeast cells, *E. coli* cells, and mouse tissues were incubated in SDS lysis buffer (4% SDS, 100 mM Tris-HCl, pH 8.5) and boiled at 95 °C for 10 min. Mouse tissues, yeast, and *E. coli* cells were subsequently homogenized using an OMNI Bead Ruptor 24 Elite instrument (6.6 m/s, 3 cycles, 30 s on, 15 s off) and sonicated using a Qsonica Q800R3 sonicator for 10 min (85% power, 10 s on, 10 s off) to shear DNA and RNA. In contrast, HEK 293T cells were directly lysed by sonication with a Qsonica Q800R3 sonicator for 10 min (85% power, 10 s on, 10 s off). Protein concentrations were quantified by measuring absorbance at 562 nm using a microplate reader, employing the Pierce BCA Protein Assay Kit (Thermo Fisher Scientific) following the manufacturer’s instructions. The lysates were aliquoted and stored at −80 °C until further use.

### ZASP

Sample volumes were adjusted based on the amount of input protein: 50 μl for 1 to 10 μg, 100 μl for 20 to 100 μg, 200 μl for 500 μg, and 500 μl for 1000 μg of input protein. Equal volumes of ZASP precipitation buffer (consisting of 0.1% formic acid and 200 mM ZnCl2 in methanol) were added to each sample, followed by a 10-min incubation at RT. Cloudiness occurred during incubation for protein inputs exceeding 5 μg. Protein pellets, not visible for inputs under 5 μg, were collected by centrifugation at 19,000*g* for 5 min at 4 °C and washed twice with an equal volume of freshly prepared methanol containing 0.1% formic acid. During washing, pellets were sonicated for 1 min using a Qsonica Q800R3 sonicator (85% power, 10 s on, 10 s off). After sonication, the methanol was evaporated at room temperature for 10 min, ensuring complete removal of residual formic acid. The resulting clean protein pellets were resuspended in 200 μl of SDC lysis buffer (1% SDC, 100 mM Tris-HCl, pH 8.5), and sonicated for 1 min to aid solubilization. Protein concentrations were measured using a BCA assay to determine the resuspended protein amount. Subsequently, TCEP (final concentration of 10 mM) and CAA (final concentration of 40 mM) were added, and the mixture was incubated for 30 min at RT for reduction and alkylation. Trypsin (Promega) was added at an enzyme-to-protein ratio of 30:1, and digestion proceeded at 37 °C for 14 h. Tryptic peptides were desalted using homemade C18 StageTips (22) and dried in a centrifugal concentrator before LC-MS analysis.

### Sample Preparation with Acetone Precipitation

The acetone precipitation method was adapted from Pena (22) and Gan (23) with some modifications. Four volumes of ice-cold acetone were added to the lysates, which were then incubated at −20 °C for 14 h. Protein pellets were obtained by centrifugation at 19,000*g* for 10 min at 4 °C. The pellets were washed once with 2.5 volumes of ice-cold acetone and twice with 2.5 volumes of ice-cold ethanol. As described in the ZASP section, the protein pellets were resuspended in SDC lysis buffer and digested with trypsin. After desalting and drying, the peptides were prepared for LC-MS analysis.

### FASP

The FASP protocol was adopted from Wisniewski (10). Protein lysates were reduced and alkylated with 10 mM TCEP and 40 mM CAA, then loaded onto a pre-equilibrated ultrafiltration spin column (Millipore, 10 kDa MWCO). The samples were washed five times with 8 M urea lysis buffer (8 M urea, 100 mM Tris-HCl, pH 8.5) and once with 50 mM ammonium bicarbonate (ABC) to remove the detergents. Proteins were digested using trypsin for 14 h at 37 °C with an enzyme-to-protein ratio of 1:30 (w/w). Peptides were collected by centrifugation and dried using a centrifugal concentrator. After resuspension in 0.1% FA, the peptides were analyzed by LC-MS.

### SP3

The SP3 protocol was described by Hughes *et al*. (3) with some modifications. Carboxylated E3 and E7 beads (Cytiva, Cat. No. 65152105050250 for E3 beads, Cat. No. 45152105050250 for E7 beads, initial concentration of 50 μg/μl) were mixed in equal proportions and washed three times with 200 μl of water. Reduced and alkylated proteins were added to the mixed beads at a ratio of 1:10 (protein to beads), ensuring the final concentration of the beads was not less than 0.5 μg/μl. Ethanol (final concentration of 80%, v/v) was used to accelerate protein aggregation, and the beads were incubated in a ThermoMixer at 1000 rpm for 10 min at RT. The supernatant was removed using a magnetic rack, and the beads were washed twice with 200 μl of 80% ethanol. The centrifuge tubes were uncapped and left at RT for 10 min to allow the ethanol to evaporate. The beads were then resuspended in 100 μl of 100 mM ABC, and trypsin was added at an enzyme-to-protein ratio of 30:1. Digestion was carried out for 14 h at 37 °C. The beads were centrifuged at 20,000*g* for 1 min, and the peptides were collected using a magnetic rack. The beads were eluted once more with 100 μl of water to increase the peptide yield. Peptides were filtered using C18 StageTips to remove the beads from the solution and dried for LC-MS analysis.

### Sample Preparation by In-Solution Digestion

The in-solution digestion protocol was adapted from Gan *et al* (23). Briefly, proteins were extracted by adding SDC buffer (1% SDC, 100 mM Tris-HCl, 10 mM TCEP, 40 mM CAA, pH 8.5) to the samples and homogenizing them with a Qsonica Q800R3 sonicator or a Bead Ruptor instrument as described in the protein extraction section. Trypsin was added at an enzyme-to-protein ratio of 30:1, and the mixture was incubated at 37 °C for 14 h. The peptides were desalted by homemade C18 StageTips and dried using a centrifugal concentrator.

### Preparation of FFPE Tissue Samples

#### Csectiolassic Method

FFPE tissues were prepared according to the methods of Coscia *et al*. (24) and Gan *et al*. (23) with some modifications. Mouse FFPE slices were soaked in xylene for 15 min to remove paraffin. The dewaxing procedure was repeated twice. Subsequently, the samples were rehydrated using a series of washes with anhydrous ethanol, 95% ethanol, 75% ethanol, and water. After rehydration, the samples were transferred to a buffer containing 300 mM Tris-HCl and 1% SDC (pH 8.5) and heated at 95 °C for 30 min for decrosslinking. Sonication was performed for 3 min using a Scientz JY-96-IIN sonicator (45 W power, 3 s on, 3 s off) to further lysis the samples. The lysate was reduced and alkylated by incubation with TCEP (final concentration of 10 mM) and CAA (final concentration of 40 mM) at RT for 30 min. Trypsin was added at a 1:30 (w/w) ratio, and digestion was carried out at 37 °C for 14 h. After desalting with C18 StageTips, the peptides were dried and prepared for LC-MS analysis.

#### ZASP for FFPE Samples

Samples were heated at 95 °C for 30 min in a buffer containing 4% SDS and 300 mM Tris-HCl (pH 8.5). The samples were then centrifuged at 19,000*g* for 10 min at 0 °C to solidify the paraffin in the top layer. The lysate and tissue debris were transferred to a new tube and sonicated in an ice water bath for 3 min using a Scientz JY-96-IIN sonicator (45 W power, 3 s on, 3 s off). One volume of ZASP precipitation buffer (consisting of 0.1% formic acid and 200 mM ZnCl_2_ in methanol) was added, and the mixture was incubated at RT for 10 min. The protein pellets were resuspended, digested, and desalted as described in the ZASP section.

### Preparation of OCT-Embedded Tissue Sections

#### Classic Method

The OCT compounds were removed as described by Holfeld (5). OCT-embedded tissues were washed twice on ice with 70% cold ethanol, then washed twice with ice-cold water and twice with 100 mM Tris-HCl. The samples were lysed in SDC lysis buffer (1% SDC, 100 mM Tris-HCl, 10 mM TCEP, 40 mM CAA, pH 8.5). Homogenization was carried out using a Bead Ruptor (OMINI, 6.6 m/s speed, 3 cycles，30 s on, 15 s off) and a Qsonica sonicator (10 min, 85% power, 10 s on, 10 s off). Trypsin digestion and desalting were performed as described in the in-solution digestion section.

#### ZASP for OCT Samples

Approximately 50 tissue volumes of 4% SDS lysis buffer were added to the OCT-embedded tissue samples. The samples were then heated at RT for 10 min and then homogenized using a Bead Ruptor (OMINI, 6.6 m/s speed, 3 cycles, 30 s on, 15 s off) and a Qsonica sonicator (10 min, 85% power, 10 s on, 10 s off). The lysates were subsequently mixed with an equal volume of ZASP precipitation buffer (consisting of 0.1% formic acid and 200 mM ZnCl2 in methanol) and incubated for 10 min at RT. Protein pellets were then washed twice with methanol solution (containing 0.1% formic acid) and resuspended in SDC. Following resuspension, proteins were digested with trypsin at a ratio of 1:30 (w/w) for 14 h at 37 °C. Peptides were desalted using C18 StageTips and stored at −80 °C prior to LC-MS analysis.

### LC-MS/MS Analysis

Peptides were subjected to analysis using an UltiMate 3000 RSLCnano liquid chromatography system (Thermo Fisher Scientific) coupled with an Orbitrap Exploris 480 mass spectrometer (Thermo Fisher Scientific). Peptides were transferred to a 30 cm analytical column (100 μm i.d., packed with 1.9 μm C18 particles) and separated at a flow rate of 300 nl/min using a 60-min gradient from 5% to 32% buffer B (acetonitrile, 0.1% formic acid). The mass spectrometer was operated in data-dependent acquisition (DDA) mode with a cycle time of 2 s. Full-MS scans were acquired at 60,000 resolution from 350 to 1500 m/z with an automatic gain control (AGC) target of 3e6 and a maximum injection time of 45 ms. Dynamic exclusion time was set to 40 s. For MS2 scan, the resolution was 15,000 at 200 m/z, with an AGC target of 3e5 and a 22 ms maximum injection time were used. Normalized HCD energy was set to 30%.

### Data Processing

The raw files were processed via MaxQuant software (version 2.0.1.0). Protein sequences retrieved from the SwissProt database (released in April 2021), encompassing entries for humans (20,387), mouse (17,098), *S. cerevisiae* (strain ATCC 204508/S288c, Baker's yeast. 6729) and *E. coli* (strain K12, 4529) sequences, were utilized. Trypsin/P was specified as the proteolytic enzyme with a maximum of 2 missed cleavage sites. Cysteine carboxamidomethylation was set as a fixed modification while methionine oxidation and protein N-terminal acetylation were considered variable modifications. The false discovery rate (FDR) was established at 1% for both peptide-spectrum matches (PSM) and protein identifications. Label-free quantification was performed using the intensity-based absolute quantification (iBAQ) method (25).

The raw DDA files from the decrosslinking experiments were processed using pFind software (26, 27) (version 3.2.0) to perform an open search for posttranslational modifications. The search parameters were configured as follows: trypsin/P was specified as the enzyme with 3 maximum missed cleavage sites. The mass tolerance for both precursor and fragment ions was set at 20 ppm. The open search mode was used, and the FDR threshold at the peptide level was 0.01. Peptides were restricted to a mass range of 600 to 10,000 Da and a length range of 6 to 10 amino acids. As per Coscia's study (24), the evaluation of decrosslinking efficiency focuses on the level of unwanted and variable chemical modifications that may affect peptide identification.

### Data Analysis

Data analysis was performed using R software (version 4.0.3) or Microsoft Excel (version 2013). The recovery rate of precipitated protein was calculated using the following formula: Protein recovery rate (%) = (precipitated protein amount)/(protein amount in lysate) ∗ 100. The precipitated protein amount = protein concentration ∗ volume of resuspension buffer. Protein concentrations were measured using a Pierce BCA Protein Assay Kit (Thermo Scientific).

For proteomic data analysis, reverse protein hits and known contaminants were first filtered out. When calculating the number of peptide or protein identifications in each sample, peptides with valid intensities (>0) and proteins with valid iBAQ values (>0) were counted. The hydrophobic “Gravy” values of the peptides were calculated using the R software "Peptides" package (version 2.4.4). For quantitative comparison, the data were log_2_ transformed and normalized by median centering. Proteins in each group with 2 missing values were filtered out. The remaining missing values were imputed via the KNN approach. Differences between 2 groups were analyzed using a two-sided *t* test with a 95% confidence interval, and *p*-values were adjusted using the Bonferroni–Holm method. Gene ontology (GO) analysis was performed using gene sets (cellular components) from the MSigDB database (m5.go.cc.v2023.1.Mm.symbols, https://www.gsea-msigdb.org/gsea/index.jsp).

### Experimental Design and Statistical Rationale

In the method development, optimization, and evaluation parts, three technical replicates were conducted to evaluate the mean of identifications, coefficient of variation (CV), and correlations. In each experiment, three technical replicates were prepared in parallel. For each comparison, samples from different groups were randomized during LC-MS measurements to ensure the elimination of the influence of LC-MS performance. For statistical analysis, t-tests were employed for two-group comparisons. The FDR was calculated based on the Benjamini-Hochberg hypothesis test, with a significance cutoff value set at 5%. When evaluating ZASP via diverse biological samples, one piece of sample from each biological type was analyzed.

## Results

### Development and Optimization of ZASP

During sample preparation, there is always a balance between the efficacy of protein extraction and the compatibility of the buffer with subsequent mass spectrometric analysis. Detergents, such as SDS, can significantly improve protein extraction from challenging samples, but most detergents are incompatible with protein digestion and LC-MS analysis. Ideally, a universal sample preparation method would include an efficacious protein extraction step and independent LC-MS analysis unaffected by lysis buffers. To develop such a universal sample preparation method for proteomics, we used zinc-assisted precipitation of proteins to link the lysis of the sample to subsequent preparation steps (Supplemental Fig. S1*A*).

**Fig. 1:**
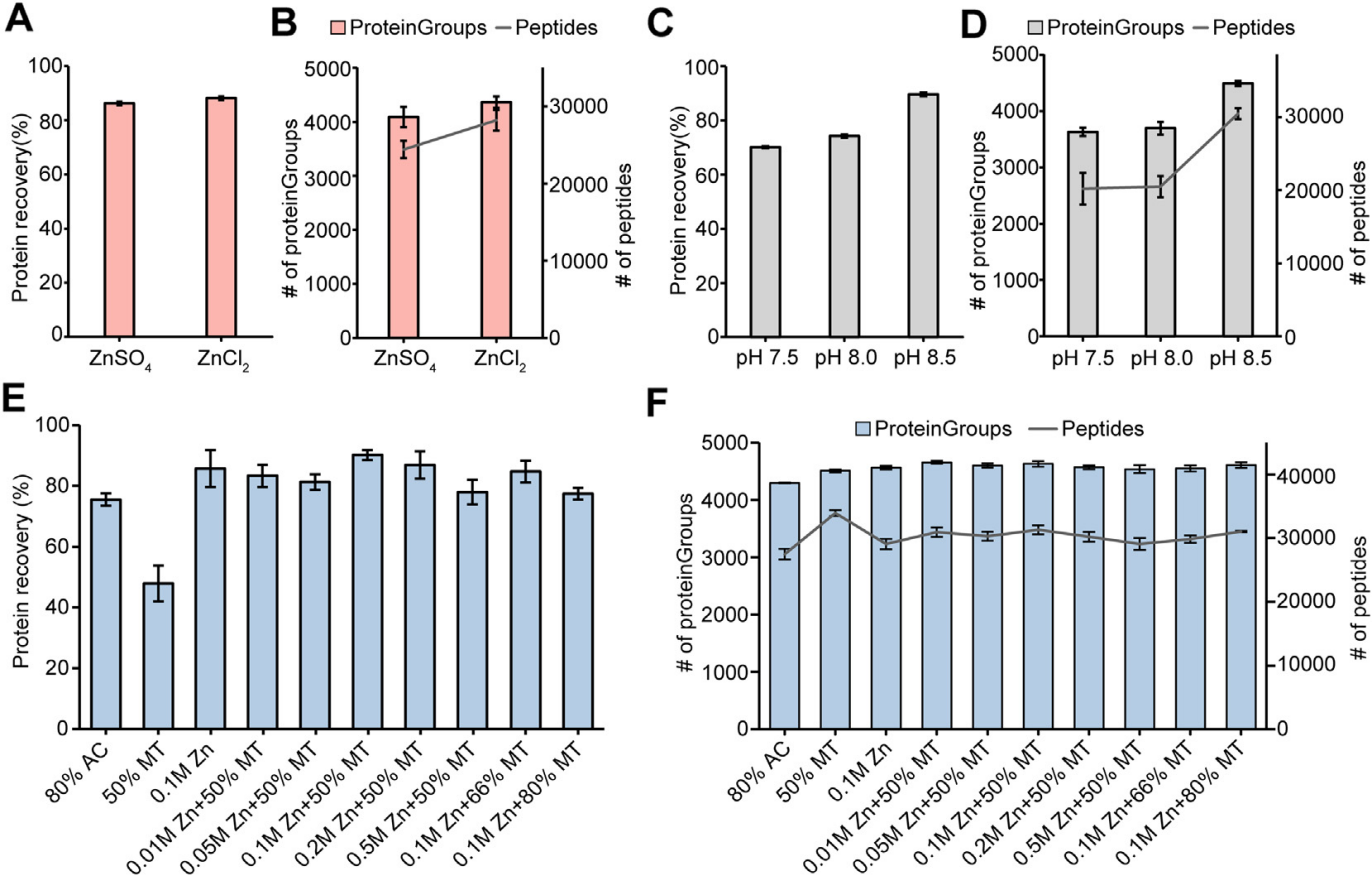
**Development and Optimization of ZASP**. *A*, protein recovery using ZASP with either ZnCl_2_ or ZnSO_4_. *B*, the number of proteins and peptides identified in HEK 293T cells using ZASP based on ZnCl_2_ or ZnSO_4_. *C*, protein recovery efficiency of ZnCl_2_-based ZASP under varying pH conditions. *D*, the number of proteins and peptides identified by ZnCl_2_-based ZASP in HEK 293T cells across different pH levels. *E*, protein recovery associated with various ZnCl_2_ and methanol mixtures. *F*, enumeration of proteins and peptides identified in HEK 293T cells treated with diverse ZnCl_2_ and methanol combinations. For comparative analyses of protein and peptide identifications, only those without missing values were considered. AC and MT denote acetone and methanol, respectively.

Zinc salts can be used for protein purification and exhibit advantages in terms of incubation time. We optimized the precipitation conditions and integrated zinc precipitation into proteomic sample preparation. Here, we assessed the effects of zinc salt type, the combination of zinc salt and organic solvent, and pH on protein recovery, the number of peptides and proteins identified, and quantification in HEK 293T cell lysates containing 4% SDS. Based on a previous study (20), we performed a comparative evaluation of 100 mM ZnCl_2_ and ZnSO_4_. The zinc salts were added to 100 μl lysate aliquots with 1 μg/μl proteins, which reached a final concentration of 100 mM. ZnCl2 and ZnSO4 precipitation showed no significant difference in protein recovery, recovery rates were 88.1% and 86.2% respectively (Fig. 1*A*). With the 60 min gradient measurement on an Exploris 480, the ZnCl_2_-based method identified 6.7% more proteins (4365 vs. 4090) and 15.5% more peptides (28,196 vs. 24,410) than the ZnSO_4_ method (Fig. 1*B*), presumably because a small number of particles could not be solubilized after the zinc sulfate precipitated. Therefore, ZnCl_2_ was chosen for subsequent method development and evaluation. Given the effect of solution pH on precipitation efficacy, we evaluated three pH values commonly used in proteomic sample preparation, including 7.5, 8.0, and 8.5. Interestingly, the protein recoveries increased with increasing pH of the lysis buffer, raised from 70.1% at a pH of 7.5 to 89.7% (Fig. 1*C*). An average of 30,483 peptides and 4489 proteins were identified at pH 8.5 (Fig. 1*D*), approximately 49.8% more peptides and 22.6% more proteins than those at pH 7.5 (20,204 peptides, 3629 proteins) and 8.0 (20,490 peptides, 3696 proteins). It indicated that pH was one of the key factors affecting sample preparation assisted by ZnCl_2_ precipitation.

For further optimization, we evaluated different concentrations of ZnCl_2_ in combination with methanol. We observed that 90.2% of the proteins were recovered by the combination of 100 mM ZnCl_2_ and 50% methanol (final concentration after mixing), which was the highest recovery. The average recovery rate was 13.2% and 42.3% greater than the acetone and methanol groups (the final concentration of methanol was 50%, v/v), respectively (Fig. 1*E*). When we precipitated proteins using ZnCl_2_ solution alone with a final concentration of 100 mM, on average 85.7% of the proteins were recovered. The increasing concentration of methanol did not help recover more proteins (Fig. 1*E*). These results suggested that ZnCl_2_ was the key factor influencing protein recovery. The comparison of different final concentrations of ZnCl_2_ showed that 100 mM ZnCl_2_ was the optimal condition. For peptide and protein identification, an average of 4543 proteins and 29,183 peptides were identified by 100 mM ZnCl_2_ and 50% methanol, whereas only 4286 proteins and 27,580 peptides were identified by acetone precipitation (Fig. 1*F*). We found no significant difference in the number of peptides or proteins identified by different combinations of ZnCl_2_ and methanol. Given the recovery of proteins and the depth of proteomic analysis, the combination of 100 mM ZnCl_2_ and 50% methanol (final concentration after mixing) was chosen as the final optimized protocol.

Taken together, the resulting ZASP consisted of protein precipitation in lysates (at pH 8.5) with ZnCl_2_ at a final concentration of 100 mM and methanol at a final concentration of 50%, protein pellet washing and resuspension, trypsin digestion and C18 StageTips desalting (Supplemental Fig. S1*A*). Precipitation can be achieved in 10 min by mixing lysates with equal volumes of ZnCl_2_ and methanol stock. The SDS was removed efficiently after 2 washes with methanol (containing 0.1% FA). To ensure effective trypsin digestion, we chose and recommended using 1% SDC buffer (1% SDC, 100 mM Tris-HCl, pH 8.5) to resuspend the proteins (Supplemental Fig. S1, *C*–*E*). Other digestion and LC-MS/MS compatible buffers, such as urea-based buffer and ammonium bicarbonate buffer, can also be used (Supplemental Fig. S1, *C*–*E*). When the protein pellets are resuspended by 1% SDC lysis buffer and reduced by TCEP, we should evaporate them in the air for at least 10 min to make sure no remaining FA (from the washing solution) in protein pellets before resuspension and prepare the TCEP solution at a neutral pH immediately before use. Otherwise, cloudiness will be observed, which is the precipitated SDC (Supplemental Fig. S1*B*).

### Performance Evaluation of ZASP

**Fig. 2:**
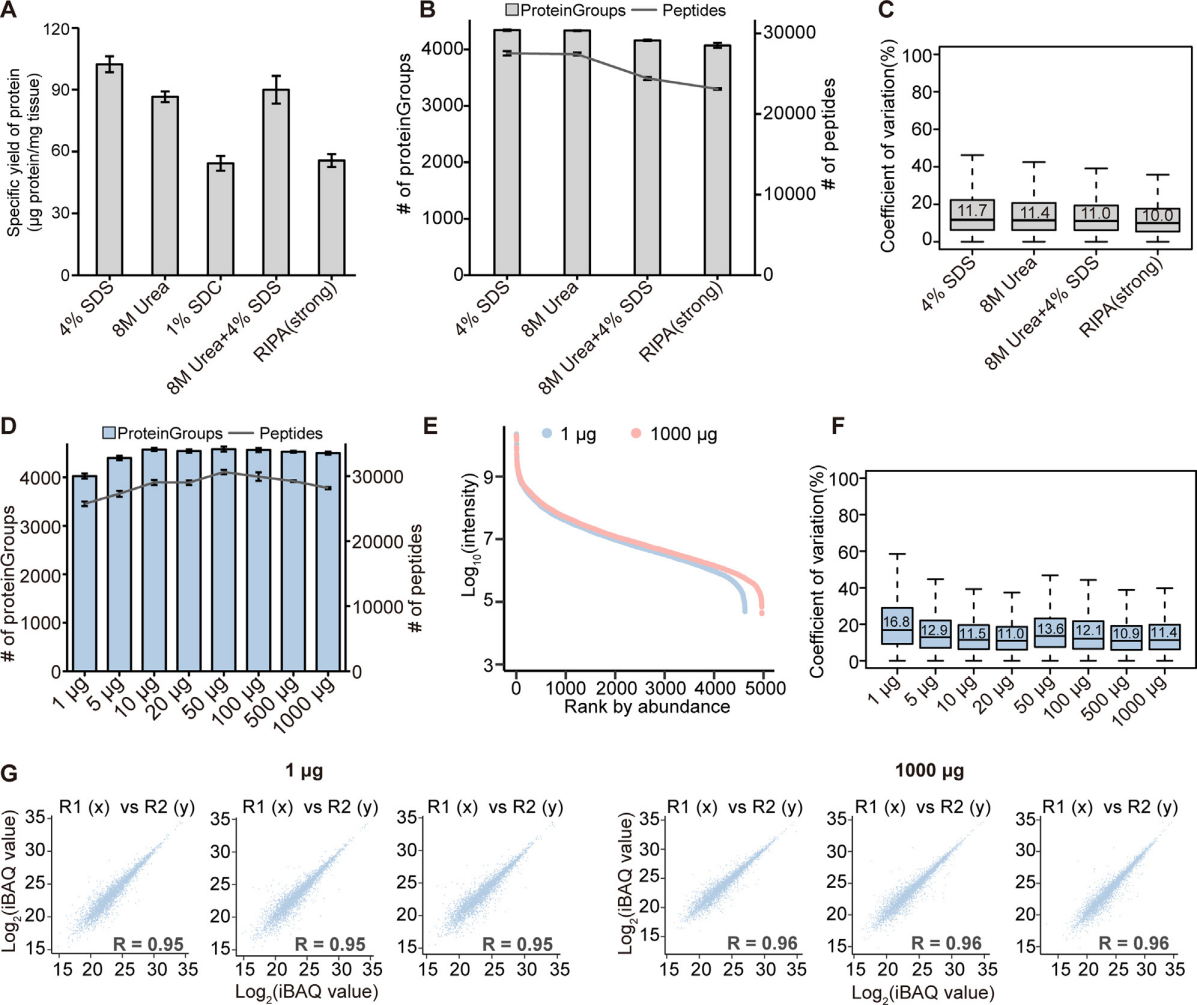
**Performance evaluation of ZASP**. *A*, comparative efficiency of various lysis buffers in protein extraction. *B*, identification of proteins and peptides in mouse small intestinal tissue using different lysis buffers. *C*, coefficients of variation for peptides associated with each lysis buffer. *D*, the number of proteins identified in mouse small intestinal tissue with protein inputs ranging from 1 μg to 1000 μg. *E*, Ranked plots illustrating protein abundance in samples with 1 μg and 1000 μg of protein input. *F*, Coefficients of variation for peptides across samples with varying protein inputs. *G*, Pearson correlation coefficients for protein identification in samples with 1 μg and 1000 μg of protein input.

SDS-based lysis buffers are widely used in proteomic analysis. The current SDS depletion strategies are time-consuming and laborious and have low reproducibility. Therefore, we developed ZASP to carry out experiments with SDS lysis buffer. To further explore its capabilities, in addition to 4% SDS lysis buffer, we also evaluated whether ZASP can be used to remove digestion/LC-MS-incompatible compounds from other commonly used lysis buffers in the laboratory, including 8 M urea, a mixture of SDS and urea, and RIPA (strong, containing Triton X-100, SDC, and SDS) buffers (Fig. 2*A*). First, we used serial sections of fresh frozen small intestinal tissues to compare the protein extraction efficacies of different lysis buffers. The 4% SDS-based lysis buffer achieved the highest protein extraction efficacy, yielding approximately 102.3 μg of protein per milligram of tissue, while 1% SDC and RIPA were much less efficient, demonstrating the superiority of the SDS lysate for protein extraction. For protein and peptide identification, 4% SDS was also superior. On average, we identified more than 4300 proteins and 27,000 peptides by 4% SDS-ZASP in a 1 h LC-MS analysis (Fig. 2*B*). The less protein extracted from tissues, the fewer peptides and proteins will be identified and quantified. For RIPA-ZASP, approximately 4000 proteins were identified. The low CVs of the quantified peptides indicated that the quantification of ZASP was highly reproducible, independent of the detergent in the lysis buffer (Fig. 2*C*). These results suggested that ZASP was highly compatible with various lysis buffers.

We next evaluated the detection sensitivity of ZASP using 1, 5, 10, 20, 50, 100, 500, and 1000 μg of mouse small intestinal proteins as input (Fig. 2*D*). For ease of handling the sample preparation procedures, we processed 1 to 10 μg of total protein per sample in a working volume of 50 μl, 20 to 100 μg of protein in 100 μl, 500 μg of protein in 200 μl, and 1000 μg of protein in a 500 μl system. As shown in Supplemental Fig. S2*A*, we recovered more than 60% and 70% of the total protein from solutions at concentrations of 0.01 μg/μl and 0.05 μg/μl, respectively. For protein concentrations ranging from 0.5 to 5 μg/μl, we were able to harvest more than 80% of the total protein. The recovery rates increased with increasing concentration of proteins in lysates. It peaked at 1 μg/μl. By 1 h of DDA analysis on an Exploris 480 instrument, ZASP identified 4037 proteins and 25,626 peptides from 1 μg of starting material. When the amount of protein input was increased to 5 μg and above, the number of proteins identified slightly increased to 4300 and peaked at 4500. The number of peptides was saturated at 28,000 to 30,000 (Fig. 2*D*). With inputs of 1 to 1000 μg of protein, the dynamic range of detected proteins spanned 6 orders of magnitude (Fig. 2*E*). The median CV of peptides in the 1 μg ZASP triplicates was 16.8%, which was relatively higher than that of the other groups, probably due to the more significant variability of protein recoveries as well as losses from pipetting. According to the DDA analysis, the overall median CV for each group was less than 20%, which demonstrated the quantitative robustness of ZASP (Fig. 2*F*). The Pearson's correlation coefficients within (>0.94) and across groups (>0.91) also confirmed its high reproducibility (Fig. 2*G* and Supplemental Fig. S2*B*). In summary, ZASP provided robust and comprehensive protein coverage for 1 μg to 1000 μg of protein input, demonstrating the good potential of ZASP for proteomic applications.

### Unbiased Evaluation of ZASP Relative to Classical In-Solution Digestion

**Fig. 3:**
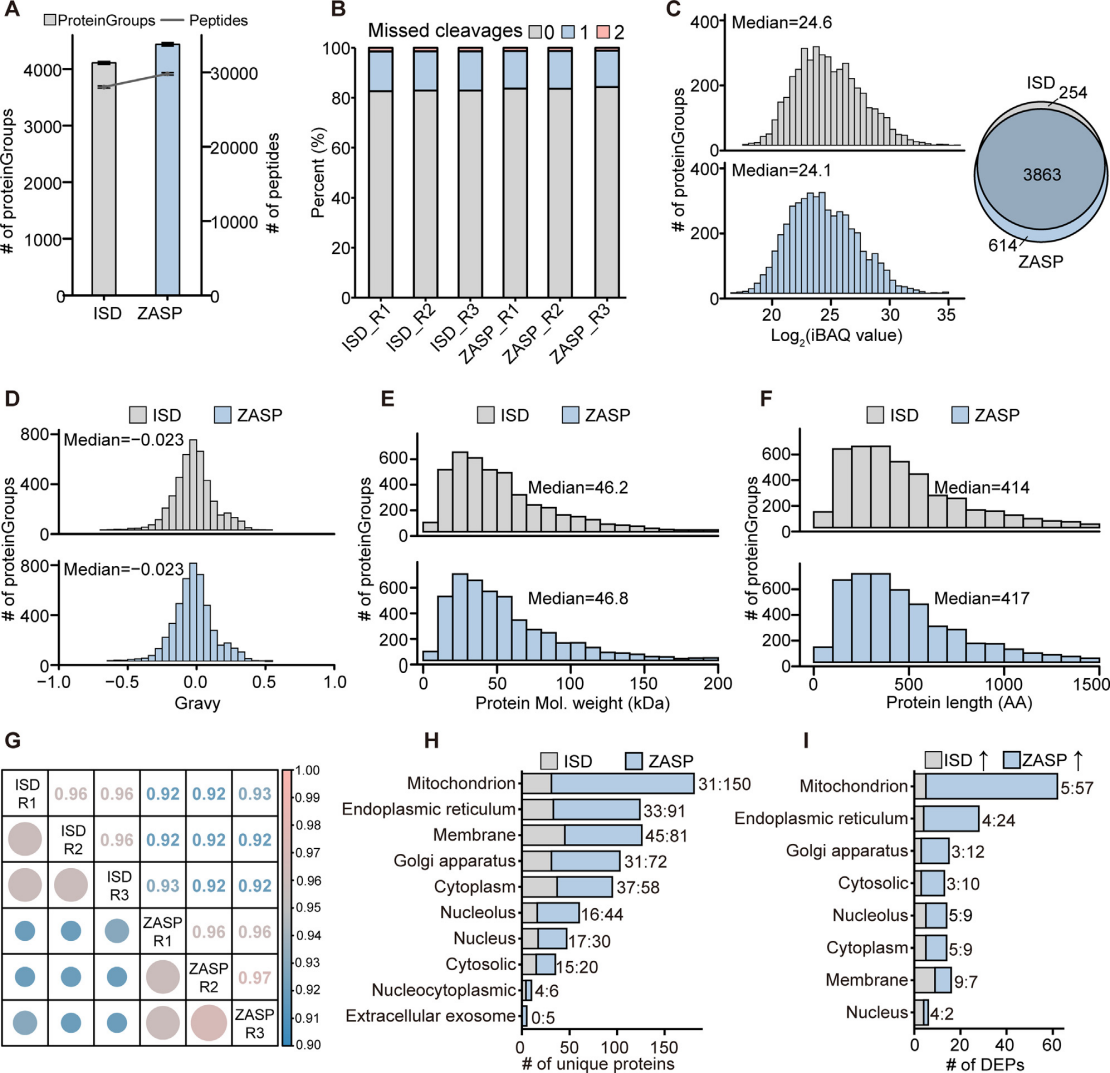
**Unbiased evaluation of ZASP relative to classical in-solution digestion.***A*, comparison of the number of proteins and peptides identified in mouse small intestinal tissue using in-solution digestion (ISD) and ZASP. *B*, missed cleavage rates of peptides identified with ISD versus ZASP. *C*, distribution of iBAQ values for proteins identified by ISD and ZASP, with a Venn diagram in the upper right corner depicting the number of common and unique proteins identified by each method. *D–F*, distribution of hydrophobicity ("Gravy" values), molecular weights, and amino acid lengths of proteins detected by ISD and ZASP. *G*, Pearson's correlation coefficients for proteins identified by ISD and ZASP. *H*, cellular component annotations for proteins uniquely identified by ISD and ZASP. *I*, cellular component annotations for shared differentially expressed proteins (DEPs) identified by both ISD and ZASP, applying Student’s *t* test (adjusted *p* < 0.05 and fold change >1.5). The number displayed in the bar graph is the number of DEPs.

Next, to determine whether the proteome identified by the ZASP method was biased, we analyzed the physicochemical properties of all identified proteins and peptides and performed a systematic comparison with a widely used in-solution digestion method, which employs 1% SDC in Tris-HCl as the lysis buffer. SDC is a mild surfactant compatible with trypsin digestion and MS analysis (28). Compared with SDC-based in-solution digestion (ISD), ZASP identified 8.0% more proteins (average ± SD, 4437 vs. 4109) and 6.3% more peptides (average ± SD, 29,800 vs. 28,025) in mouse small intestinal tissue samples (Fig. 3*A*). Of the 4117 proteins and 27,822 peptides identified by ISD, ZASP detected 3863 proteins and 20,630 peptides (Fig. 3*C* and Supplemental Fig. S3*A*). Incomplete removal of SDS can severely affect trypsin digestion efficacy and decrease identification rates. Here, ZASP produced a very similar proportion of missed cleavages as ISD (the mean percentage of no missed cleavages was 83.9% vs. 82.8%), indicating that SDS was efficiently depleted and that trypsin activity was unaffected (Fig. 3*B* and Supplemental Fig. S3*B*).

Since ZASP is a protein-level precipitation-based method, it is necessary to determine whether proteins with specific characteristics are missing. As shown in Figure 3, *D*–*F*, the hydrophilicity, molecular weight, and amino acid length distributions of the proteins identified by ZASP and ISD were similar. At the peptide level, the distributions of hydrophilicity, peptide length, charge, and mass were also similar, whereas ZASP produced more peptides with 10 to 20 amino acids and more peptides with a 2^+^ charge (Supplemental Fig. S3, *C*–*F*). ZASP can rapidly recover proteins from the solution without causing significant bias. The correlation coefficients of protein abundance between biological replicates and any two different samples were extremely high (Pearson's coefficient ≥0.92), suggesting high reproducibility of the two methods (Fig. 3*G*).

Annotation analysis revealed that proteins identified only by ZASP were enriched in membrane-bound organelle proteins, mainly organelles such as mitochondria (150 of 614), the endoplasmic reticulum (91 of 614), and the Golgi apparatus (72 of 614) (Fig. 3*H*). By comparing the abundance of common proteins identified by both methods, we identified a relatively small set of proteins with a fold change ≥1.5 and an FDR≤0.05, a total of 160 proteins (Supplemental Fig. S3*G*). 118 proteins with intensities higher than those of the ISD group were obtained by ZASP. Similar to the unique proteins identified by ZASP, 102 of these proteins were from membrane-bound organelles, and 57 of these proteins were from mitochondria (Fig. 3*I*). The 254 unique proteins and 42 proteins with higher intensities identified in the ISD analysis were also enriched in membrane-bound organelle proteins (Fig. 3, *H* and *I*), which may be due to differences in the properties of the SDS and SDC. In summary, the shared proteins quantified by ZASP and SDC-ISD are highly consistent; meanwhile, ZASP can increase the identification of proteins from membrane-bound organelles (especially mitochondria) and improve the depth of analysis benefiting from the high protein extraction efficiency of SDS.

### Comparative Analysis of ZASP and Other Detergent-Depletion Sample Preparation Techniques

**Fig. 4:**
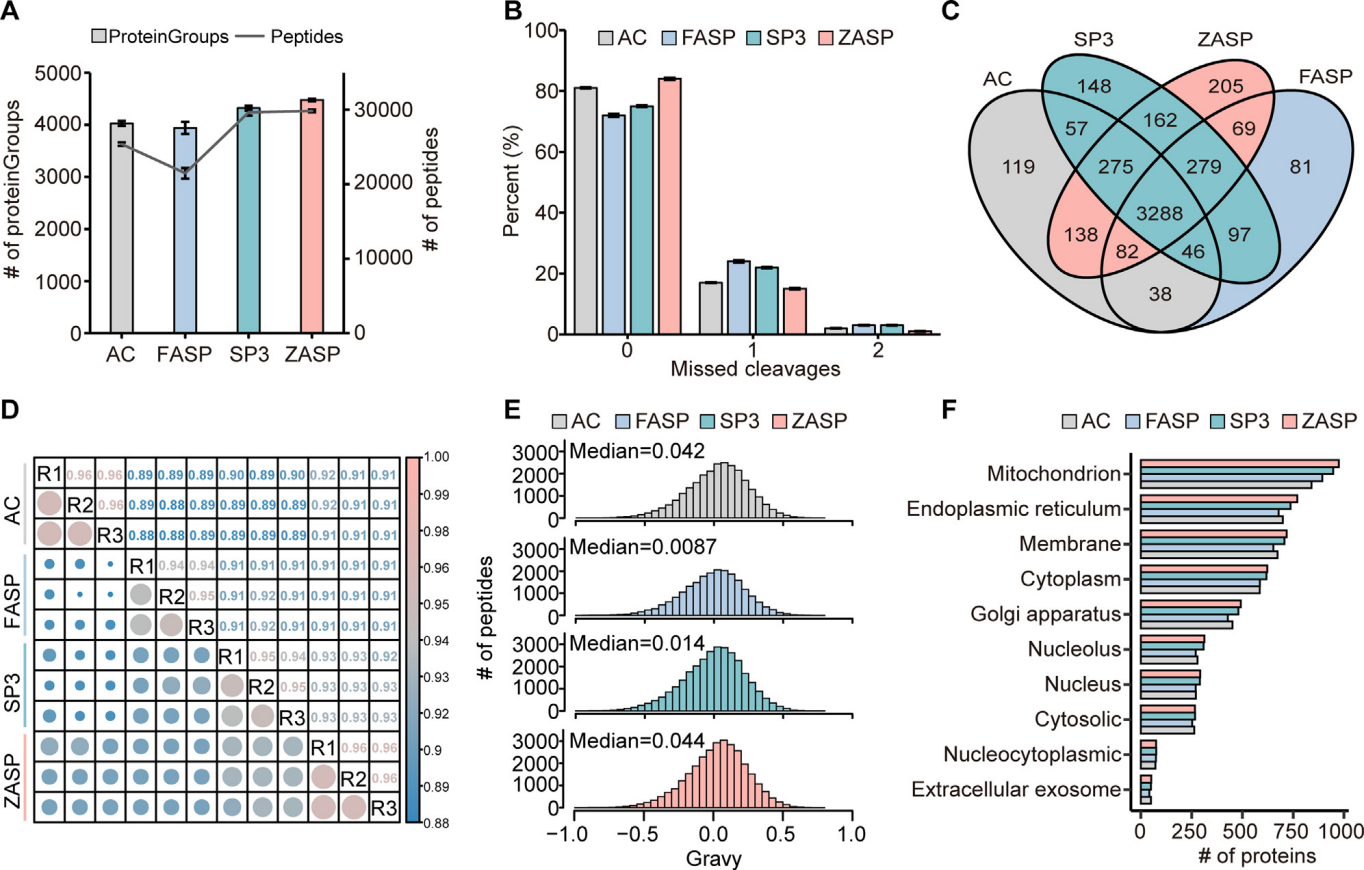
**Comparative analysis of ZASP and other detergent-depletion sample preparation techniques**. *A*, the number of proteins and peptides identified using acetone precipitation (AC), FASP, SP3, and ZASP methods. *B*, missed cleavage rates of peptides identified by each of the four methods. *C*, Venn diagrams illustrating the overlap of proteins identified by AC, FASP, SP3, and ZASP. *D*, Pearson's correlation coefficients for protein identifications across AC, FASP, SP3, and ZASP. *E*, distribution of hydrophobicity ("Gravy" values) of peptides identified by AC, FASP, SP3, and ZASP. *F*, cellular component annotations for proteins identified by AC, FASP, SP3, and ZASP.

Next, we compared ZASP with the acetone precipitation, FASP, and SP3 methods, the three most commonly used mass spectrometry incompatible detergent removal strategies in proteomic analysis. Acetone precipitation is a widely used method for recovering proteins from different solutions and can be easily linked to subsequent in-solution digestion proteomic workflows. FASP is a filter-assisted proteomic sample preparation method that removes incompatible reagents by exchanging buffers. SP3 is a sample preparation method that uses nanoparticles to aggregate proteins and is compatible with a wide range of detergents. As shown in Figure 4*A*, ZASP identified an average of 4456 proteins (n = 3) in the mouse small intestinal samples, 11.0% and 13.2% greater than the numbers identified by the acetone precipitation and FASP methods, respectively. At the peptide level, ZASP detected 17.6% and 39.0% more peptides than acetone precipitation (29,871 *vs.* 25,404) and FASP (29,871 *vs.* 21,494), respectively. ZASP identified a few more proteins and peptides than SP3 without significant difference (proteins: 4474 *vs.* 4319, peptides:29,871 *vs.* 29,660). ZASP generated the most significant proportion of peptides without missing cleavages: 83.7% by ZASP, 80.9% by acetone precipitation, 72.4% by FASP, and 75.0% by SP3 (Fig. 4*B*). The higher missing cleavage rates of FASP could be explained by the incomplete removal of SDS caused by insufficient buffer exchange during ultrafiltration (5 times with 500 μl of urea buffer in this study), which also lead to less peptide and protein identifications (Fig. 4, *A*–*C*). Although SP3 identified very similar numbers of peptides and proteins to ZASP, it suffered higher missed-cleavage rates due to the less exposure of enzymatic sites on proteins, which was supported by Wang *et al*. (29) and Dieters-Castator *et al*. (30). The percentage of shared proteins among the four methods was 64.7% (Fig. 4*C*), which indicates consistency in protein identification among the different methods. The Pearson correlation coefficients at the protein level were 0.96 across ZASP replicates, 0.92 to 0.93 between ZASP and SP3, and 0.91 to 0.92 between ZASP and FASP or acetone precipitation methods, indicating high reproducibility of ZASP and quantitative consistency between ZASP and other detergent-depletion sample preparation methods (Fig. 4*D*). Moreover, the hydrophilicity, molecular weight and amino acid length distributions of the proteins or peptides detected by the ZASP method were almost identical to those detected by the acetone precipitation, FASP and SP3 methods (Fig. 4*E* and Supplemental Fig. S4, *A* and *B*). Gene Ontology analysis based on cellular components for proteins identified by each method showed that the number of proteins localized to the top 10 cellular locations was similar across all four methods (Fig. 4*F*). This suggested that ZASP did not introduce processing biases compared to other well-established and widely used methods.

### ZASP Analysis of Diverse Biological Samples

**Fig. 5:**
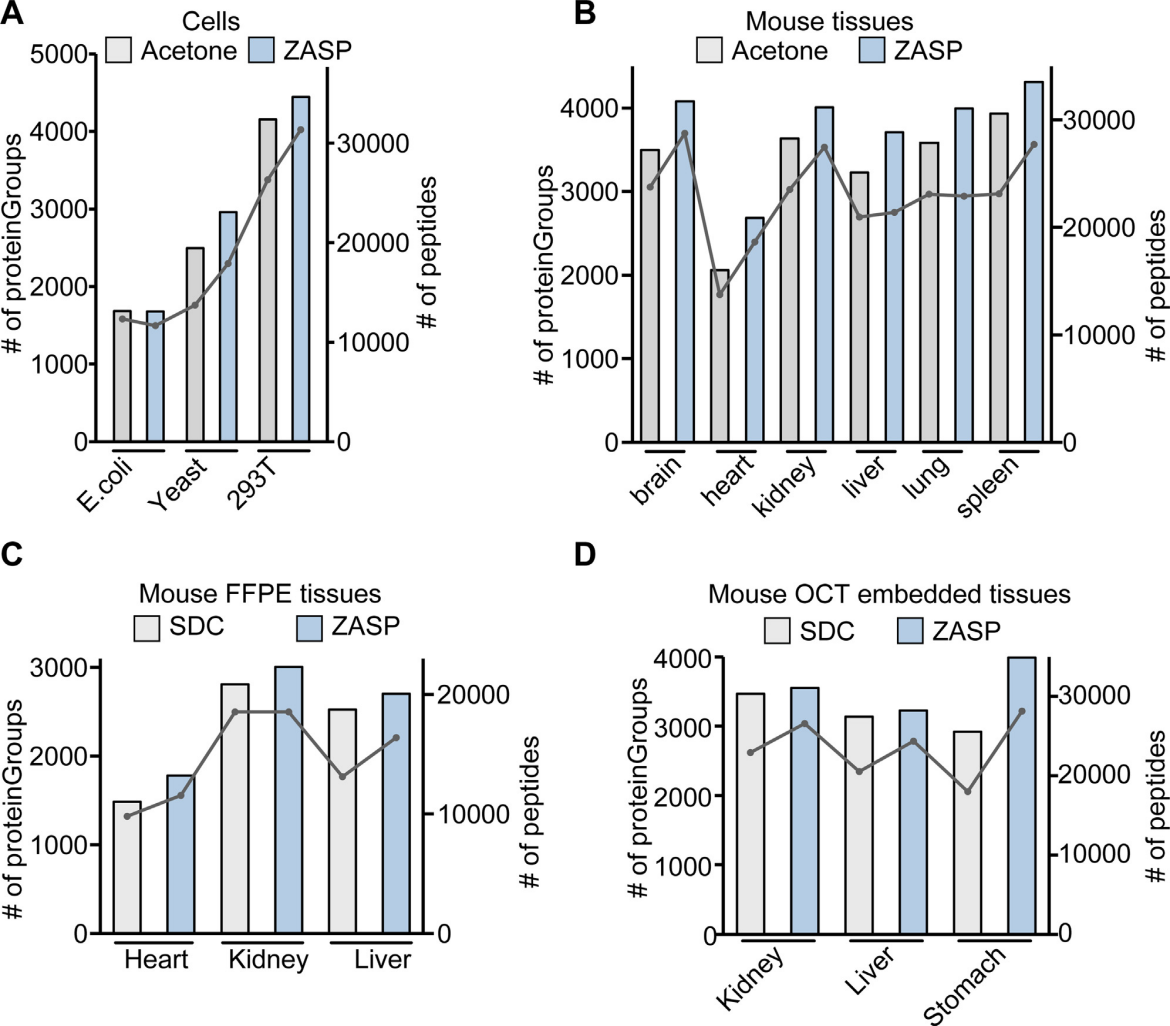
**ZASP analysis of diverse biological samples**. *A*, the number of proteins and peptides identified across different cell types. *B–D*, enumeration of proteins and peptides identified in mouse fresh frozen tissues, formalin-fixed paraffin-embedded (FFPE) tissues, and tissues embedded in optimal cutting temperature (OCT) compound.

To analyze the performance of ZASP in different applications, in addition to HEK 293T cells and mouse small intestinal tissue, yeast, *E. coli*, fresh frozen mouse tissues, and FFPE and OCT-embedded samples were processed with ZASP. Denatured yeast, *E. coli*, and frozen tissues were homogenized with a bead disrupter in SDS lysis buffer. As shown in Figure 5*A*, we characterized the *E. coli* proteome at almost the same depth as that of the acetone precipitation method, whereas the percentage of yeast and HEK 293T cell proteins identified by the ZASP method increased by 18.6% and 7.0%, respectively. According to the 1 h DDA analysis of 6 different fresh frozen tissues, ZASP improved the proteomic depth and coverage, especially in the brain and heart. The number of proteins and peptides detected by ZASP in the brain increased by 16.6% and 21.1%, respectively (Fig. 5*B*). These results demonstrated that ZASP is a promising method for preparing different cell and tissue samples.

For FFPE and OCT samples, removing paraffin and OCT compounds is a critical factor in determining the success of the analysis. Traditional FFPE sample preparation procedures require dewaxing with xylene, rehydrating the tissue with an ethanol gradient, and decrosslinking by boiling it in an alkaline solution (23). Multiple washes with ethanol have been reported to be an effective method for removing OCT compounds (5, 31, 32). However, these extra steps increase complexity, timing, and sample loss, making the analysis especially challenging for size-limited FFPE and OCT-embedded samples. Here, we developed streamlined protocols for these two difficult sample types using ZASP (Supplemental Fig. S5*A*). FFPE slices were boiled in SDS lysis buffer (pH 8.5) for 30 min and centrifuged at 0 °C to separate the paraffin. Then, we added the ZASP precipitation stock solution to harvest the proteins, followed by in-solution digestion. For OCT-embedded samples, the tissues were lysed directly in the SDS buffer, and the other steps were the same as those for the ZASP method described above. Compared to the xylene-based method, FFPE-ZASP identified 1781, 3007, and 2705 proteins in the heart, kidney, and liver, signifying increases of 19.6%, 7.0%, and 7.2% in each organ. (Fig. 5*C*). Moreover, this protocol provided effective decrosslinking, and the percentages of lysine methylation and methionine oxidation were less than 9.0% (Supplemental Fig. S5*B*), similar to the classic approach and the study of Fabian *et al* (24). For OCT-embedded samples, ZASP also achieved deeper measurements than SDC-based in-solution digestion with sequential ethanol washing, particularly in the stomach samples (Fig. 5*D*). The number of identified proteins increased from 2920 to 3989. We only measured limited samples in this section, so ZASP might not always provide more comprehensive measurements for all sample types. Here, we have shown that ZASP can significantly simplify the sample processing procedure for challenging sample types without sacrificing performance.

## Discussion

Comprehensive, accurate, and deep proteomic analysis requires the reproducible generation of peptides from the entirety of proteins present in each sample. Harsh denaturants, such as SDS, are required for total protein solubilization from difficult matrices. Unfortunately, even at low concentrations, detergents can preclude enzymatic digestion and contaminate mass spectra. In this work, we demonstrated that ZASP effectively depletes detergents and other interfering impurities in lysates before trypsin digestion. The combination of ZnCl_2_ and methanol allows effective precipitation of proteins in the lysates in 10 min at room temperature. After the excess SDS was removed, the protein pellets were resuspended and processed straightforwardly by in-solution digestion. The reaction in small volume resulting from the mixing of an equal volume of precipitation solution and cell or tissue lysates significantly decreases sample loss. ZASP shows advantages in processing time, flexibility, and scalability. It simplifies sample preparation effectively and can be directly applied to diverse sample types. ZASP will be an attractive approach for the proteomics community.

The performance assessment of ZASP for analyzing samples with varying detergents and sample inputs revealed that this method is highly flexible and robust. ZASP can effectively remove SDS, Triton X-100, and urea at high concentrations and recover proteins from solutions with a wide range of protein concentrations. The presence of 1% to 4% SDS is sufficient for total protein solubilization in diverse samples, including hard tumor tissues, and is routinely used in biochemical studies (10, 23, 33). Tissue samples lysed with 4% SDS-based lysis buffer (4% SDS, 100 mM Tris-HCl, pH 8.5) performed better than those treated with the other buffers in recovering the most significant amount of protein and quantifying the greatest number of proteins. Therefore, we chose 4% SDS as the lysis buffer for all the evaluation experiments and recommended it as the standard protocol for performing ZASP. Moreover, ZASP has shown exceptional performance in analyzing a wide range of protein starting amounts (ranging from 1 μg to 1000 μg), as evidenced by similar analysis depth and strong reproducibility. This capability makes ZASP suitable for use in comprehensive proteome analysis with limited samples and in the analysis of posttranslational modifications (PTMs) where a large amount of protein is required for enrichment.

Compared to SDC-based in-solution digestion, acetone precipitation, FASP, and SP3, ZASP exhibited superior performance in several aspects. SDC is an anionic detergent for protein extraction and can be removed by adding formic acid before LC-MS analysis. SDC (1%) has also been reported to increase the digestion efficacy of trypsin (34). Encouragingly, the percentage of peptides without missed cleavages generated via ZASP was comparable to that of SDC-ISD, again providing evidence of the effective depletion of SDS. The similar distributions of protein molecular weight and hydrophobicity as those of SDC-ISD confirmed the unbiased precipitation of ZASP. Upon utilizing the SDS lysis buffer, ZASP demonstrated enhanced protein extraction efficacy in comparison to SDC-ISD. Acetone precipitation is generally used to recover proteins in solutions, which requires cold (−20 °C) acetone, a volume four times that of the protein samples, and incubation for more than 12 h at −20 °C (20, 35). In contrast, ZASP simply requires a trivial amount of time (10 min) and labor at RT and provides higher protein recoveries than acetone precipitation. FASP is designed to remove SDS and excess reagents during proteomic sample preparation by utilizing a molecular weight cutoff (MWCO) membrane as a ‘reactor’. FASP suffers sample loss when processing a low microgram range of samples, and variations are caused by incomplete removal of SDS (36). ZASP outperforms FASP in the completeness of impurity removal and reproducibility and can accommodate various protein inputs. SP3 is an increasingly popular sample preparation method, utilizing a single reaction vessel, carboxylate-modified magnetic beads, and organic solvent-induced protein aggregation to exchange or remove contaminants in lysates before digestion. However, several steps can easily cause sample losses and variability, for example, incomplete protein aggregation on magnetic beads and incomplete bead disruption during wash steps. SP3 steps are required to be strictly followed (3). Besides, it is not friendly for PTM analysis (which needs larger protein inputs) because of protein loss and bead costs (37). ZASP precipitates proteins via cost-effective and commonly used reagents that can be easily accessed in most laboratories. ZASP allows us to handle a wide range of input proteins (from 1 to 1000 μg) and lysates with a wide range of protein concentrations (the total protein concentration can be as low as 0.01 μg/μl) and ensures the depth of analysis in the meantime. ZASP is incredibly simple, requiring only commonly used reagents, incubation, centrifugation, and pipetting.

ZASP can be easily applied to analyze samples from various biological sources, including those presenting challenges. For FFPE and OCT-embedded specimens, we integrated ZASP to streamline conventional workflows. The optimized workflows offer efficacious extraction by SDS, followed by the complete removal of SDS, paraffin, and OCT compounds via one-step precipitation. ZASP simplified the FFPE and OCT slice analyses and enabled comprehensive proteomic profiling. Because of these inherent benefits, ZASP has the potential to be widely adopted in the proteomic community as a rapid, low-cost, and universal sample preparation method. Furthermore, ZASP can theoretically be scalable to a 96-well format and can be adapted to automated workstations to achieve high throughput and implement multiomics in a single sample by recovering proteins from DNA and RNA extraction leftover. Future developments of ZASP-based approaches will facilitate proteomic research.

## Data Availability

The mass spectrometry proteomic data and MaxQuant database search results have been deposited to the ProteomeXchange Consortium (http://proteomecentral.proteomexchange.org) *via* the iProX partner repository (38, 39) with the dataset identifier PXD042276.

## Supplemental data

This article contains supplemental data.

## Conflicts of interest

The authors declare that they have no conflicts of interest with the contents of this article.
