# Supplementary material for: ZASP: A Highly Compatible and Sensitive ZnCl2 Precipitation-Assisted Sample Preparation Method for Proteomic Analysis
Source: Mol Cell Proteomics. 2024 Sep 6;23(10):100837. doi: 10.1016/j.mcpro.2024.100837 (PMC11492125; doi:10.1016/j.mcpro.2024.100837)
Supplement: Supplemental FIgures [file mmc1.pdf]

Supporting information

ZASP: A highly compatible and sensitive $ZnCl_2$-precipitation assisted sample preparation method for proteomic analysis

Xianfeng Shao[1, 2, 3, 4], Yuanxuan Huang[1, 2, 3, 4], Rong Xu[5, 1], Qiqing He[3, 4], Min Zhang[6], Fuchu He[1, 2, 3, 4, 5*] and Dongxue Wang[1, 2, 3, 4*]

[1] State Key Laboratory of Proteomics, Beijing Proteome Research Center, National Center for Protein Sciences (Beijing), Beijing Institute of Lifeomics, Beijing 102206, China

[2] Beijing Proteome Research Center, Beijing 102206, China

[3] International Academy of Phronesis Medicine，Guangzhou 510005，Guangdong, China

[4] The π-Hub Infrastructure，Guangzhou 510535，Guangdong, China

[5] Guangzhou Laboratory, Guangzhou 510005, Guangdong, China

[6] Department of Dermatology, Xiangya Hospital of Central South University, Changsha, 410008, Hunan, China

[*]Correspondence to:

Fuchu He: hefc@bmi.ac.cn

Dongxue Wang: wang_dongxue@126.com

Supplemental Figures

Figure S1: Establishment of the ZASP Method.

Figure S2: Evaluation of ZASP sensitivity.

Figure S3: Comparison of physicochemical properties of proteins identified by ISD and ZASP.

Figure S4: Comparison of ZASP with AC, FASP, and SP3.

Figure S5: Characterization of diverse biological samples prepared by ZASP.

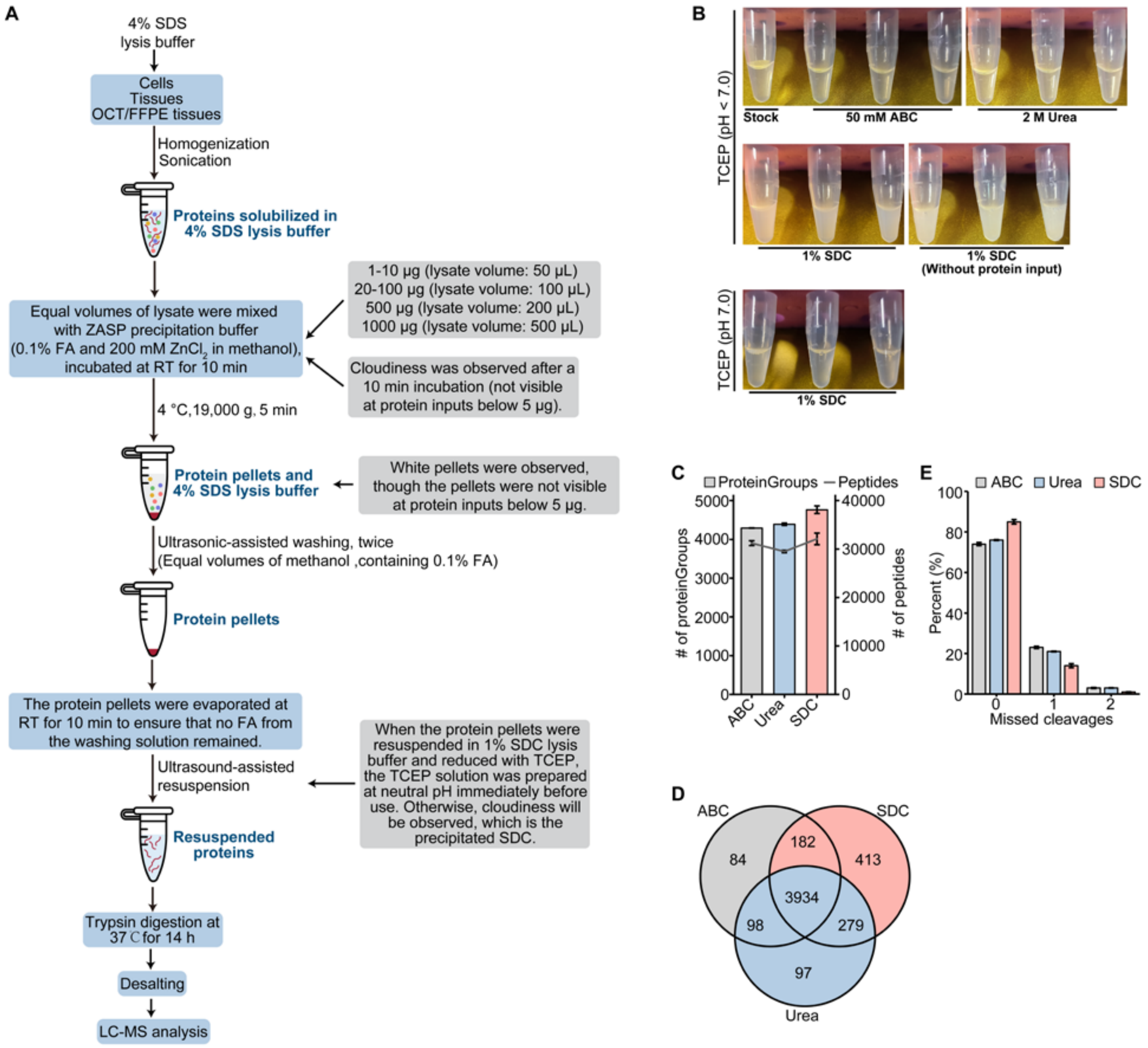

Figure S1. Establishment of the ZASP Method. (A) Workflow depicting the steps involved in ZASP. (B) Comparison of various buffers for protein resuspension. (C) The number of proteins and peptides identified using different buffers for resuspending protein particles. (D) The overlap of proteins resuspended in different buffers. (E) Missed cleavage rates of peptides identified with different buffers for resuspending protein particles.

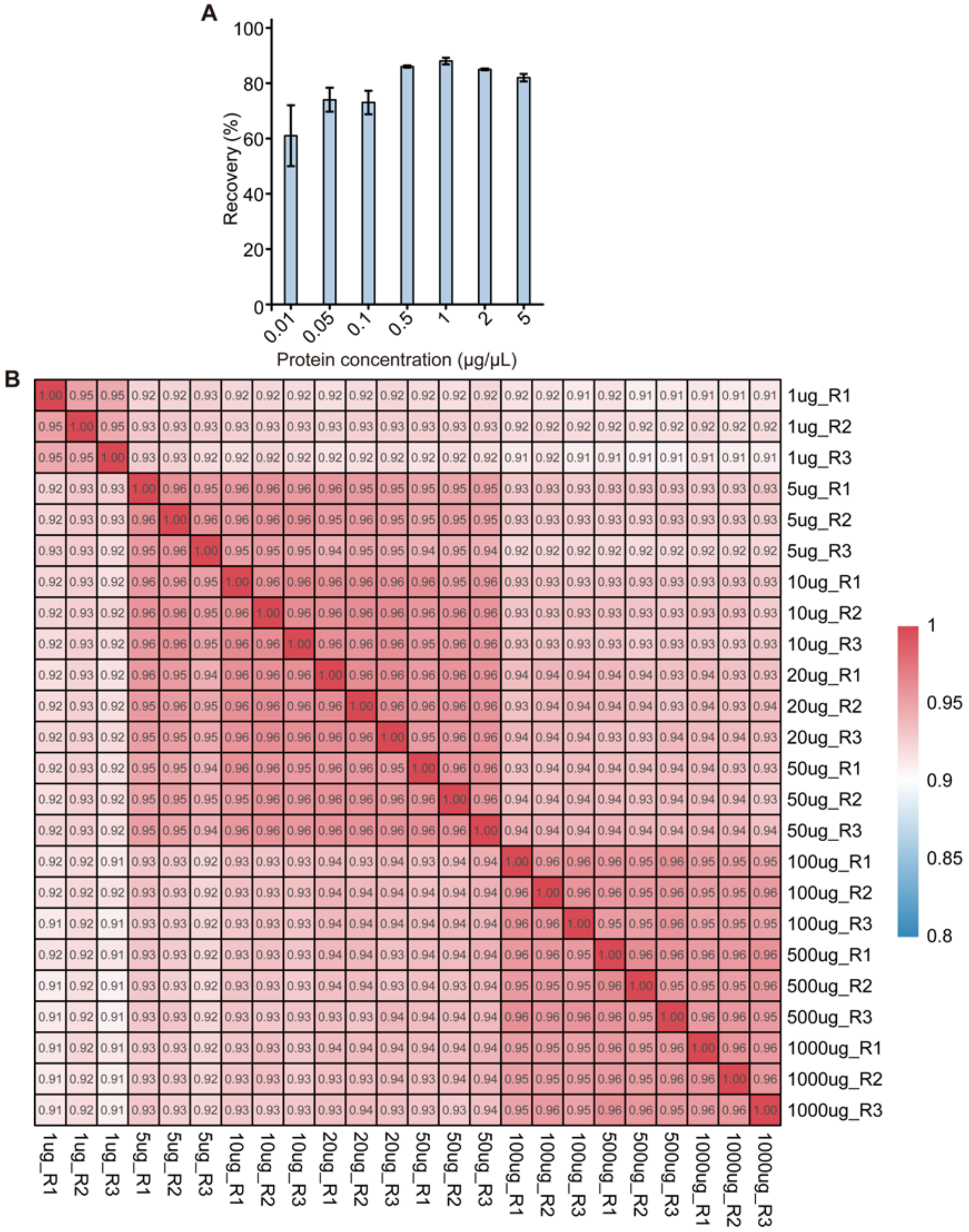

Figure S2. Evaluation of ZASP sensitivity. (A) Protein recoveries of ZASP in mouse small intestinal tissues at various protein concentrations. (B) Pearson's correlation coefficients of proteins in mouse small intestinal tissue across protein inputs ranging from 1 μg to 1000 μg.

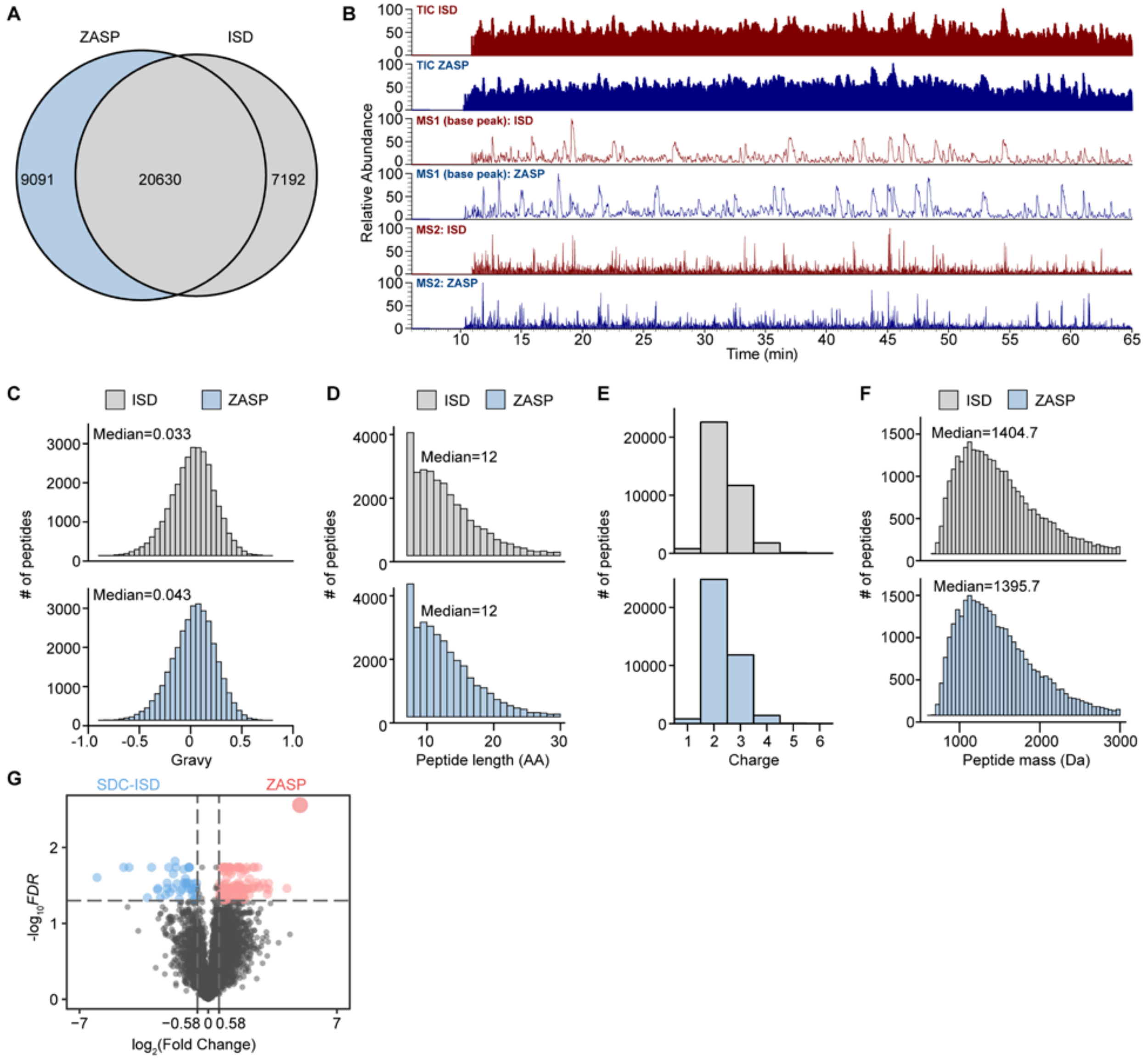

Figure S3. Comparison of physicochemical properties of proteins identified by ISD and ZASP. (A) Overlap of peptides identified by ISD and ZASP. (B) Comparison of total ion current (TIC), base peak (MS1), and MS2 profiles between ISD and ZASP. (C-F) Distribution of "Gravy" values, amino acid lengths, charges, and molecular weights of peptides identified by ISD and ZASP. (G) Volcano plot showing the differences between proteins commonly identified by ISD and ZASP.

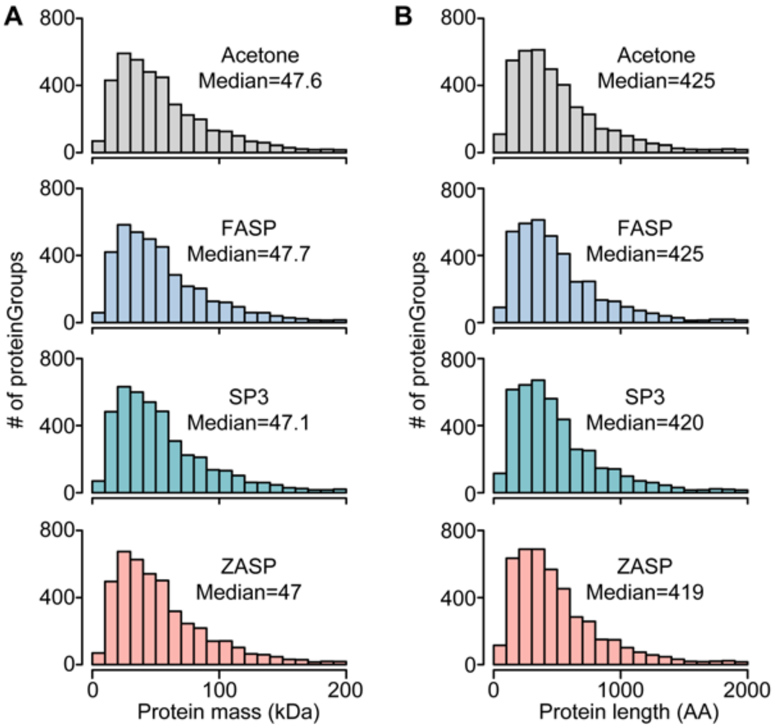

Figure S4. Comparison of ZASP with AC, FASP, and SP3. (A-B) Comparative analysis of molecular weights and amino acid lengths of proteins identified by AC, FASP, SP3, and ZASP.

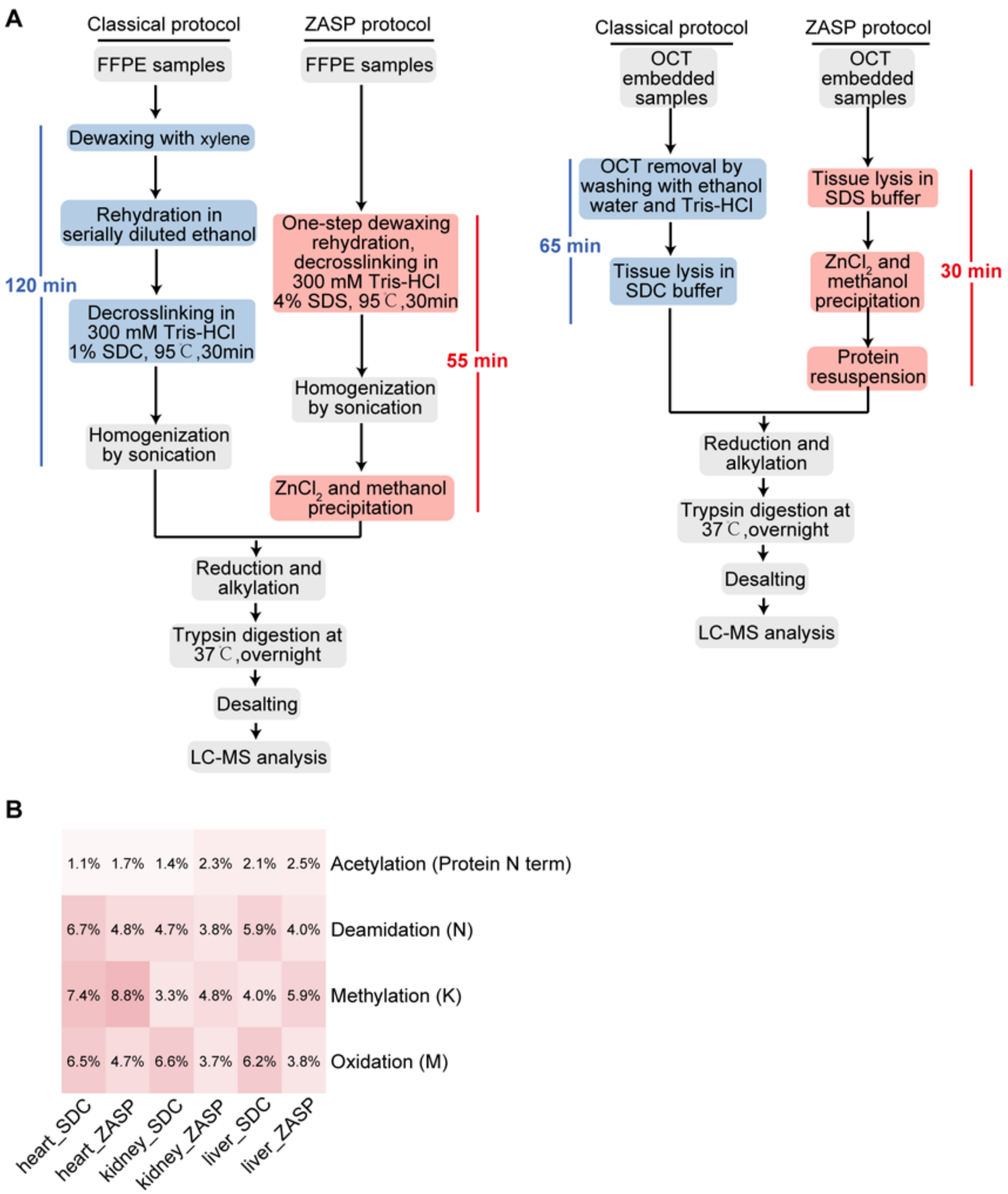

Figure S5. Characterization of diverse biological samples prepared by ZASP. (A) ZASP workflow for the preparation of formalin-fixed paraffin-embedded (FFPE) and optimal cutting temperature (OCT)-embedded samples. (B) Assessment of the decrosslinking efficiency of ZASP for FFPE sample preparation.
